# Rapid Pyrazinamide Drug Susceptibility Testing using a Closed-Tube PCR Assay of the Entire *pncA* gene

**DOI:** 10.1038/s41598-020-61286-7

**Published:** 2020-03-06

**Authors:** Michael G. Whitfield, Salvatore A. E. Marras, Rob M. Warren, Annelies Van Rie, John Rice, Lawrence J. Wangh, Barry N. Kreiswirth

**Affiliations:** 1 South African Medical Research Council Centre for Tuberculosis Research, DST-NRF Centre of Excellence for Biomedical Tuberculosis Research, Division of Molecular Biology and Human Genetics, Stellenbosch University, Stellenbosch, South Africa; 2 Public Health Research Institute, Rutgers University, Newark, New Jersey, United States of America; 3 Department of Epidemiology and Social Medicine, Faculty of Medicine, University of Antwerp, Antwerp, Belgium; 4 Department of Biology, Brandeis University, Waltham, Massachusetts, United States of America; 5 Center for Discovery and Innovation, Nutley, New Jersey, United States of America

**Keywords:** Biochemistry, Biological techniques, Molecular biology, Diseases, Medical research, Molecular medicine

## Abstract

The continued use of pyrazinamide in the treatment of tuberculosis in the absence of a rapid, accurate and standardized pyrazinamide drug susceptibility assays is of great concern. While whole genome sequencing holds promise, it is not yet feasible option in low resource settings as it requires expensive instruments and bioinformatic analysis. We investigated the diagnostic performance of a closed-tube Linear-After-The-Exponential (LATE)-PCR assay for pyrazinamide susceptibility in *Mycobacterium tuberculosis*. Based on a set of 654 clinical *Mycobacterium tuberculosis* culture isolates with known mutations throughout the *pncA* gene as determined by Sanger sequencing, the assay displays excellent sensitivity of 96.9% (95% CI: 95.2–98.6) and specificity of 97.9% (95% CI: 96.1–99.7). In a subset of 384 isolates with phenotypic drug susceptibility testing, we also observed high sensitivity of 98.9% (95% CI: 97.5–100) but lower specificity of 91.8% (95% CI: 87.9–95.8) when compared to phenotypic drug susceptibility testing. We conclude that the LATE PCR assay offers both a rapid and accurate prediction of pyrazinamide susceptibility.

## Introduction

### Background

Pyrazinamide (PZA) is an integral component of the treatment regimens for drug susceptible and drug resistant tuberculosis (TB) that are currently recommended by the World Health Organization (WHO)^1–4^. Moreover, PZA will likely remain an important anti-TB drug in the future as it is included in multiple ongoing clinical trials for drug susceptible and drug resistant TB due to its unique synergistic properties with new TB drugs (pretomanid and bedaquiline)^5,6^. In light of the high rates of PZA resistance (39% − 60%) in patients with multi-drug resistant (MDR)-TB and extensively drug resistant (XDR)-TB^7,8^, rapid assessment of PZA susceptibility is important to enable clinicians to select ≥ 4 effective antibiotics to increase chance of treatment success. Nevertheless, drug susceptibility testing (DST) for PZA is rarely performed because it requires stringent control of pH and inoculum size^9–11^ and is therefore unreliable in most settings^12–15^.

Several studies have demonstrated that numerous mutations in the *pncA* gene are the primary cause of resistance to PZA^16–18^. It is hypothesized that these variants cause decreased PZase activity, thereby limiting the conversion of PZA to the active form of pyrazinoic acid (POA) within *M. tuberculosis*^19^. DNA sequencing has identified over 600 different insertion, deletions and single nucleotide polymorphisms (SNPs) across the entire length of the *pncA* gene, making identification of resistance causing mutations in this gene extremely challenging using methods other than sequencing the entire *pncA* gene^8,20–23^. Sequencing is therefore regarded as essential to detect all possible variants associated with PZA resistance and involves either Sanger sequencing of a PCR amplified *pncA* gene product^24^, targeted deep sequencing, or whole genome sequencing (WGS)^25^. Three recent large studies assessed the performance of molecular assays to predict PZA drug susceptibility^26–28^. A study using targeted sequence data observed an overall sensitivity of 49% and specificity of 98%^26^. One WGS studies achieved similar specificities but higher sensitivity of 65%^27^. When also including low-frequency SNPs a sensitivity of 91% was achieved.

WGS currently requires a culture isolate, making this approach unrealistic for routine laboratories in most high burden countries. The closed-tube Linear-After-The-Exponential (LATE)-PCR assay offers a novel and promising alternative. To detect all possible sequence variations in the *pncA* gene, the LATE-PCR^29^ method efficiently generates an abundance of one full length *pncA* gene target strand over the other and employs Lights-On/Lights-Off probes^30^ in the same closed-tube. LATE-PCR is a non-symmetric PCR method that uses a Limiting Primer and an Excess Primer whose initial melting temperature adhere to the equation Tm_0_^L^ − Tm_0_^X^ ≥ 0. This simple rule guarantees efficient amplification of double strands, followed by efficient amplification of a single strand when the Limiting Primer runs out. The Lights-On/Lights-Off probes are comprised of sets of dual-labelled fluorescent probes and adjacent quencher-only probes that coat the entire target sequence when the reaction temperature is dropped at end-point. The unique sequence of the underlying target is revealed by the temperature-dependent pattern in which the Lights-On and Lights-Off probes melt off when the temperature at end-point is slowed increased. This approach allows for highly reliable and robust detection of mutations in a target amplicon in the absence of any form of sequencing and without opening the tube.

LATE-PCR and Lights-On/Lights-Off probes have already been used before to detect mutations in the *rpoB*, *katG*, *inhA promoter* genes/regions responsible for resistance to rifampicin and isoniazid in *M. tuberculosis*^31^. The present report demonstrates for the first time that LATE-PCR can be used to efficiently amplify the GC-rich target *pncA* gene that is 685 nucleotides long. Lights-On/Lights-Off probe pairs can be designed in standardized sets comprised of shorter Off-probes and longer On-probes whose placement along the single-stranded target sequence does not need to be fitted to the target sequence, which may or may not fold into temperature-dependent hairpins. In addition, the innovative probe design used here did not use the three different colors to partition the target into separate sections, as described previously^30,31^. Instead, probes labelled with Quasar 670, Cal Red 610, and Cal Orange 560 are interspersed along the length of the target with the result that the fluorescent signature in each color is a composite fluorescent signature of separate positions along the length of the target.

## Results

### Comparison of PZA susceptibility using Sanger sequencing vs lCosed-Tube Assay

**Figure 1:**
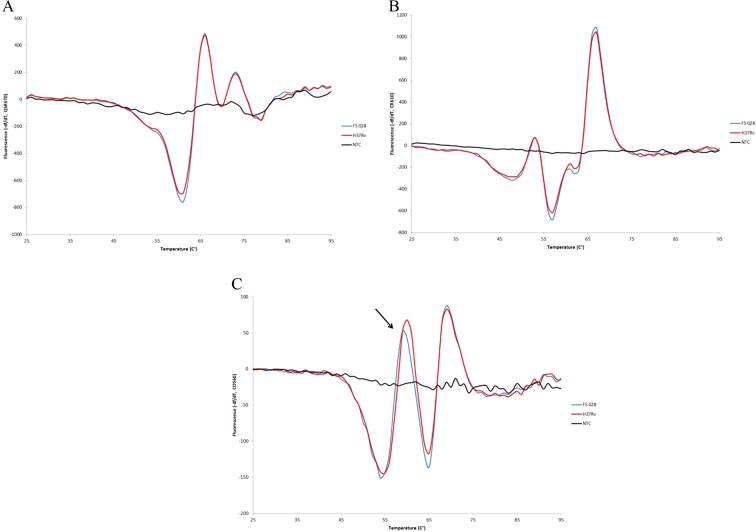
(**A**) Lights-On/Lights-Off probe fluorescence signature for Quasar 670. This figure shows the first derivative of the fluorescence profile for Quasar 670. The red fluorescence signature is the control of H37Rv; the blue fluorescence signature is the test/unknown sample and the black fluorescence signature is the no template control (NTC). No observed difference in this figure. (**B**) Lights-On/Lights-Off probe fluorescence signature for Cal Fluor Red 610. This figure shows the first derivative of the fluorescence profile for Cal Fluor Red 610. The red fluorescence signature is the control of H37Rv; the blue fluorescence signature is the test/unknown sample and the black fluorescence signature is the no template control (NTC). No observed difference in this figure. (**C**) Lights-On/Lights-Off probe fluorescence signature for Cal Fluor Orange 560. This figure shows the first derivative of the fluorescence profile for Cal Fluor Orange 560. The red fluorescence signature is the control of H37Rv; the blue fluorescence signature is the test/unknown sample and the black fluorescence signature is the no template control (NTC). We observe a shift in the fluorescence signature (indicated at the arrow), revealing that this strain harbors a variant.

Examples of the fluorescence signatures for wildtype and variants in the different fluorescent colors are shown in Fig. 1, along with additional signatures in supplemental data (supplemental figures). In Fig. 1A and B we observe fluorescent signatures for the reference strain and the test strain to overlap in Q670 and CR610, however the fluorescent signature shifts by 1 °C around the 60 °C in CO560 (Fig. 1C) which suggests a variant is present in the test strain compared to the reference strain. Supplemental table 2 lists the *pncA* genotypes detected using Sanger sequencing, the PZA phenotypes, and the Closed-Tube Assay Scores for all isolates. Among the 654 *Mtb* isolates, Sanger sequencing identified 239 (36.5%) as wild type *pncA* sequences while the Closed-Tube Assay scored 234 isolates as wildtype, for a concordance of 97.9%. Sanger sequencing identified 415 (63.5%) as *pncA* variant sequences. Among a total of 181 different *pncA* mutations, the genetic alteration included: insertions (n = 20), deletions (n = 18), SNPs (n = 123), promoter SNPs (n = 7), and double SNPs (n = 13). Although the Closed-Tube Assay does not distinguish these different classes of mutations, its concordance with Sanger sequencing for overall mutation detection was 97.2% (636/654). Of the 415 isolates identified as mutations by sequencing, 402 (96.9%) were also classified as *pncA* variants by the Closed-Tube Assay.

Overall the comparison of Sanger sequencing and the Closed-Tube Assay demonstrate that the novel approached used here had a sensitivity of 96.9% (95% CI: 95.2–98.6) and specificity of 97.9% (95% CI: 96.1–99.7) for the detection of *pncA* variants.

### Phenotypic PZA DST vs Closed-Tube detection for determination of PZA susceptibility

Among the isolates there was a subset of 384 for which pDST results were available: 188 (48.9%) were resistant to PZA and 196 (51.1%) were susceptible to PZA. The Closed-Tube Assay identified 202 (52.6%) of these isolates as *pncA* variants and 182 (47.4%) as *pncA* wildtype. Thus, the overall concordance between the Closed-Tube Assay and the pDST culture method was 95.3% (366/384). Of the 196 phenotypically PZA susceptible isolates, 180 (91.8%) were also classified as PZA susceptible by the Close-Tube Assay. Of the 188 phenotypically PZA resistant isolates, 186 (98.9%) were correctly classified as drug resistant by Closed-Tube Assay. Thus, diagnosis of PZA resistance using the Closed-Tube Assay has a sensitivity of 98.9% (95% CI: 97.5–100) and specificity of 91.8% (95% CI: 87.9–95.8).

## Discussion

**Figure 2:**
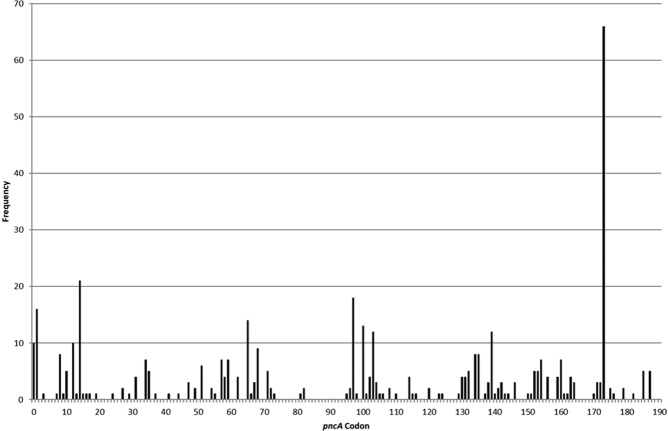
p*ncA* variant frequency. This figure shows the distribution of different *pncA* variants, covering the entire *pncA* gene, as well as the frequency of these *pncA* variants, which were present in the cohort of 654 clinical *Mtb* isolates.

The results presented here demonstrate that the closed-tube method that was previously described for the detection of mutations with relatively short amplicons from the *rpoB, katG* and *inhA* promotor regions^31^ is a flexible technology that can be adapted for detection of virtually all sequence variants in a large set of purified *Mtb* DNA isolates from patients in South Africa and the United States. Moreover, these sequence variants are spread throughout the full length *pncA* gene target of 685 nucleotides. The strains tested included *pncA* SNPs widely distributed across the entire *pncA* gene (Fig. 2), providing a good representation of the distribution of mutations observed globally^8^. The performance of the Closed-Tube Assay was excellent, with a sensitivity of 96.9% and specificity of 97.9% compared to *pncA* Sanger sequencing, and a sensitivity of 98.9% and specificity of 91.8% when compared to pDST testing. The assays utility was not affected by the position in the gene that was analysed. The observed performance is similar to that reported for Sanger sequencing of the *pncA* gene, a line probe-based assay (Genoscholar PZA-TB II line probe assay, NIPRO Corporation, Japan) and WGS^24,32^. The line probe assay showed high 97.6% concordance with a composite reference standard *pncA* Sanger and Illumina sequencing plus phenotypic susceptibility testing. A *pncA* Sanger sequencing study observed a sensitivity of 90.9% and specificity were of 100% when using pDST as the reference standard^24^. Both, Sanger sequencing and pDST require additional steps, which are not required in the close-tube method. The Sanger sequencing, while returning a result within 48 hours, still requires a sequencing instrument. The line probe-based assay avoids the need for a sequencing instrument, but both assays require transfer of DNA (opening the tube) either to load onto a sequencer or for hybridization onto a strip membrane, this creates the opportunity for environmental contamination. The sensitivity and specificity achieved using the closed-tube approach are comparable to what was observed in the 100,000 genomes project (91% and 97% sensitivity and specificity), but did not require the use of costly WGS^28^. The cost of goods of this single tube assay is under $0.50, this includes the plastics, primers, probes, and PCR reagents, but excludes the cost for DNA extraction and culture of the isolate.

Of the 18 isolates which showed discordant results, 5 were scored as *pncA* wildtype using Sanger sequencing, but showed a shift in their fluorescent signatures using the Closed-Tube Assay and 13 contained a *pncA* variant using Sanger sequencing, but showed no shift in fluorescent signatures. Of the 5 isolates scored a wildtype on Sanger sequencing, 4 had changes in the Cal Fluor Red 610 fluorophore channel. The fluorescent signature shifts can be detected down to 90:10 mixtures of different genotypes (unpublished data). Thus, 4 of the 5 isolates scored as wildtype by Sanger sequencing may have been due to the presence of hetero-resistance that is below the level of detectability by Sanger sequencing^33,34^. The *pncA* variants in isolates where no shift in the fluorescent signatures were not clustered and observed between codon 27 and codon 187 as well as in the promoter region. It is also possible that there is a shift in the fluorescent signatures that is extremely subtle and is not detected.

The excellent sensitivity and specificity coupled with a rapid turnaround time for a result (same day), and relatively inexpensive costs of the reaction components means that the Closed-Tube method holds promise for a diagnostic to identify *pncA* mutations. Nevertheless, there are some limitations of the study described here. First, the samples used in this study were purified DNA, which requires culturing for 4–6 weeks as well as DNA extraction. This could be resolved by validating the assay using a “raw” specimen type such as the Hain Lifescience FluoroLyse Kit for Genomic Bacterial DNA (Hain Lifescience, GmbH, Nehren, Germany) from both sputum as well as culture. This would demonstrate the ease of use at point of care, where no culturing is required. Second, while we evaluated an extensive list of different clinical *pncA* mutations the full spectrum of mutations has not been evaluated. Finally, the assay focuses on any mutation in the entire *pncA* gene, which may result in identification of mutations not associated with PZA resistance^26,35,36^. The next version of the assay could be expanded to include targets from the *rpsA* gene, since mutations in this gene have been associated with PZA resistance and to develop an algorithm to relate specific mutations to fluorescent signatures for *pncA* mutations not associated with PZA resistance^37,38^.

In summary, we have successfully designed and tested a single Closed-Tube Assay that is able to identify and distinguish the wildtype *pncA* gene from strains with *pncA* sequence variants that cause PZA resistance. This assay is for detection of PZA resistance accurate and rapid, and relatively inexpensive compared to both genotyping by sequencing and phenotyping by culture. Additional refinements, including use of PCR thermal cyclers that are more precise than the Stratagene MX3005p, will improve identification of the rare synonymous mutations and the few non-synonymous mutations in *pncA* that were not correctly scored as PZA resistance. This virtual sequencing method is a promising advance in novel diagnostics for PZA and could be integrated alongside the GeneXpert (Cepheid, Sunnyvale, CA, USA) or Line Probe Assay to determine the patient’s resistance profile so that the treatment can be optimised. Parallel comparisons using these different methods would likely also improve understand of resistance to PZA, which is such an important drug in the fight against tuberculosis.

## Methods

### Ethics statement

Methods and protocols were carried out in accordance with the relevant guidelines and regulations. The isolates used from South Africa was approved by the Health Research Ethics Committee of Stellenbosch University (S12/01/020). The isolates used from the Center for Discovery and Innovation were selected from an archived collection of isolates all of which are de-identified.

### Clinical isolates

Clinical *Mycobacterium tuberculosis* (*Mtb*) culture isolates (n = 654) were selected from a culture bank at Stellenbosch University (n = 424) and from the global *Mtb* strain collection archived at the Center for Discovery and Innovation (n = 230). Isolates were selected to have as many different *pncA* variants as possible and where possible biological replicates of different *pncA* variants as well as wild type. DNA was purified from each isolate as per the procedure described by Warren *et al*.^39^. The *pncA* gene was Sanger sequenced for all samples according to the method of Streicher *et al*.^24^.

The clinical isolates from Stellenbosch University included 115 RIF-mono resistant; 38 poly-resistant; 158 MDR-TB and 113 XDR-TB isolates. Unfortunately, the drug resistant profile was not available for the clinical isolates from the archived collection at the Center for Discovery and Innovation.

Phenotypic susceptibility to PZA was performed using the non-radiometric BACTEC MGIT 960 method as per the manufacturer’s instructions. This system makes use of modified test media to support the growth at a pH of 5.9. To distinguish between resistant and susceptible isolates, a critical concentration of 100 µg/ml PZA is utilized (BD Diagnostic Systems, NJ, USA). PZA drug susceptibility testing (DST) results were available from a subset of isolates, 384 of 654 (59%).

### Closed-tube assay for detection of *pncA* gene variants

LATE-PCR^29,40,41^ primers (pncA_LP3: 5’ – GCGGCGTCATGGACCCTATATCTGTGGCTGCCGCGTC – ‘3 and pncA_XP3 5’ – TTGCTCCACCGCCGCCAACAGTTCAT – ‘3) were designed to amplify a 685 bp amplicon encompassing the entire coding region of the *pncA* gene, the 37 bp of the upstream sequence and 24 bp of the downstream sequence. In total, 17 pairs of Lights-On/Lights-Off probes were designed, according to Rice *et al*. protocol^30^, to cover the entire *pncA* gene including upstream and downstream flanking regions (Supplemental table 1). Lights-On probes of between 20–26 bp were designed to be complementary to a distinct region of the single-stranded DNA of *pncA* generated by the LATE-PCR. The primers and probes were designed using Visual-OMP software (DNA Software Inc., Ann Arbor, MI, USA). Each Lights-On probe is a low-temperature dual-labelled molecular beacon with a two base pair self-complementary stem and was labelled with a fluorophore on its 5’end and a Black Hole quencher on its 3’end. Each of the 17 Lights-Off probes serve to absorb energy from the fluorophore of its adjacent Lights-On probe when both are bound to the target strand. Lights-Off probes of 15 bp were labelled with a Black Hole Quencher I (BHQ-1) or Black Hole Quencher 2 (BHQ-2) quencher. Some probes included nucleotide mismatches to their wildtype target sequences to adjust the probe melting temperature. Probes designed in this way will nevertheless change Tm when hybridized to a target sequence that includes a mutation.

**Figure 3:**
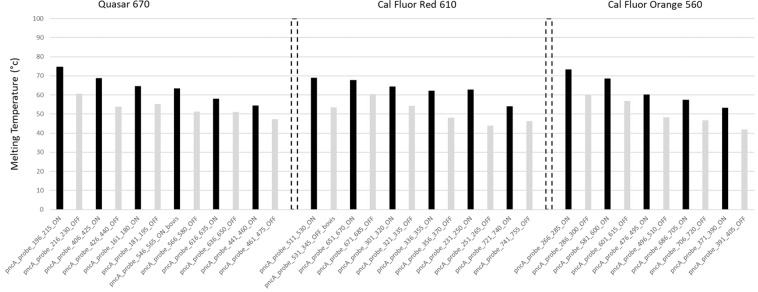
Lights-On/Lights-Off probe placement on the coding region of the *pncA* gene. *pncA* probes in the three fluorescence colors showing each probes respective Tm. The dashed bars separate different fluorescent colors (Quasar 670; Cal Fluor Red 610; Cal Fluor Orange 560). The black bars indicate the ON probes while the grey bars indicate the OFF probes. The probe name indicates the gene (*pncA*), the nucleotide position in the *pncA* gene, and lastly whether the probe is an ON or OFF probe.

The probes were catalogued into three groups according to their fluorophore labels (Quasar 670, Cal Fluor Red 610 and Cal Fluor Orange 560) (Fig. 3). Multiple pairs of probes of the same color or different colors can be used to analyse sequences several hundred nucleotides long. During development the probes Tm and binding were checked utilizing Visual-OMP software (DNA Software Inc., Ann Arbor, MI, USA). Each fluorescent contour was transformed into its first derivative curve, which we refer to as a fluorescent signature^30^. This design results in a unique fluorescent signature for each probe target sequence, making it possible to detect the presence or absence of any nucleotide change in the *pncA* gene and flanking regions.

PCR amplification of extracted *Mtb* DNA was carried out using a Stratagene MX3005p device (Agilent Technologies, Santa Clara, CA, USA). Each reaction mixture (25 μl) contained 1 to 2.5 μl of template DNA (purified DNA 25 ng/ μl), 0.05 μM of the pncA_LP3 and 1.0 μM of the pncA_XP3 PCR primers, 1× PCR buffer, 3.5 mM magnesium, 0.6 μM of PrimeSafe^40^ (Smiths Detection, London, UK), 0.3 mM deoxynucleoside triphosphates, 0.05 μM of each of the 34 Lights-On probes and 0.15 μM of each of the 34 Lights-Off probes, 25 nM Internal Marker labelled with Quasar 670, 75 nM Internal Marker labelled with BHQ-2, and 0.125 μl (1.5 U) of Taq polymerase (Thermo Fisher Scientific, MA, USA). All reagents were added in a PCR preparation room. The PCR mix was then taken to another clean room where the DNA (up to 25 ng/µl) for each sample was added under sterile conditions and the tube sealed to minimize the risk of contamination. Water was included as a negative control and H37Rv DNA as a positive control. The amplification protocol consisted of 60 cycles of 98 °C for 10 seconds and 75 °C for 40 seconds, followed by a final elongation step of 75 °C for 10 minutes. After amplification, the temperature of the reaction was lowered to 25 °C for 10 minutes. This results in the coating of the single-stranded DNA templates with 17 Lights-On/Lights-Off fluorescent probe pairs labeled in three colors. The temperature was then increased from 25 °C to 95 °C at a rate of 1 °C for 30 seconds while concurrently measuring fluorescence. Fluorescent signatures were compared to the signature of a pan-susceptible strain (H37Rv). Deviations in the signature pattern were interpreted (visual inspection) to reflect the presence of genetic variants in the *pncA* gene as they would alter the melting temperature of the complementary probe.

### Statistical analysis

The sensitivity and specificity of the virtual sequencing method were determined with R: Foundation for Statistical Computing, https://www.R-project.org (Vienna, Austria).

## Supplementary information

Supplementary Data.

## Data Availability

The datasets generated during and/or analysed during the current study are available from the corresponding author on reasonable request.
